# Salivary cortisol in university students after the COVID-19 pandemic

**DOI:** 10.1016/j.cpnec.2022.100160

**Published:** 2022-09-20

**Authors:** Nicole Andelic, Julia Allan, Keith A. Bender, Ioannis Theodossiou, Daniel Powell

**Affiliations:** a Centre for European Labour Market Research, Economics Department, Business School, University of Aberdeen, Aberdeen, United Kingdom; b Scottish Experimental Economics Laboratory, Economics Department, Business School, University of Aberdeen, Aberdeen, United Kingdom; c Health Psychology, Institute of Applied Health Sciences, University of Aberdeen, Aberdeen, United Kingdom

**Keywords:** Stress, Cortisol, COVID-19

## Abstract

The COVID-19 pandemic required people to navigate lockdowns and unfamiliar restrictions for the first time. It is known that situations characterised by uncontrollability and novelty heighten the physiological response to stress. The data presented here was collected as part of an experimental stress study and offered an opportunity to compare cortisol levels upon arrival to the lab before and after the first UK lockdown, when students had to navigate novel health and safety restrictions on campus.

Participants (*n* = 152) were students who took part in an experiment designed to measure salivary cortisol levels as a response to a stress task. All provided a baseline cortisol sample after arriving to the lab but before the experimental task. Pre-lockdown participants (*n* = 72) were familiar with the campus rules whereas post-lockdown participants (*n* = 80) had to adhere to novel restrictions, including health questionnaires, PPE and social distancing.

The post-lockdown sample had significantly higher levels of baseline cortisol, cortisol output (AUCg) and cortisol response (AUCi) than the pre-lockdown group. This effect remained significant even after controlling for sample characteristics.

These findings suggest that navigating new restrictions may lead to heightened levels of anticipatory stress even if there is no difference in recent general mental health before and after the lockdown.

## Highlights

• We compared salivary cortisol on arrival to an experiment in participants post-lockdown with those attending pre-lockdown.
• The post-lockdown participants had higher levels of baseline cortisol, AUCg and AUCi, than those attending pre-lockdown.
• These differences remained even after controlling for sample characteristics.

## 1 Introduction

The COVID-19 pandemic became a rapidly growing threat to public health after its outbreak in 2019. In addition to the relatively high mortality rate, several studies have already established that COVID-19 had a substantial psychological impact on mental health. For example, large-scale surveys have already found that at the height of the pandemic approximately 20% of respondents in China experienced symptoms anxiety or depression [1,2] and prevalence of mental distress increased from 18.9% to 27.3% in the UK population [3]. This negative impact on mental health is perhaps not surprising considering the severity of the virus and the disruption to daily life as many countries enforced national restrictions during the height of the pandemic [4].

Since March 2020, there have been various iterations of lockdowns or restrictions, not entirely removed in the UK until Spring 2022. Even though these restrictions were put in place as a safety measure, navigating the novel restrictions may also have been the source of some stress. Indeed, multiple surveys of hospital staff find that in addition to fears about contracting or spreading COVID-19, major workplace stressors included wearing PPE and adhering to restrictions in the work environment [5,6]. However, we do not know whether these restrictions led to a physiological stress response.

A number of studies have examined the relationship between the COVID-19 pandemic and cortisol. Heightened cortisol concentration is associated with working from home or job loss in mothers [7] as well as stress in nurses [8] during the peak of the pandemic. Furthermore, during the pandemic lockdown, perceived stress was predicted by pre-pandemic cortisol awakening response (CAR) moderated by resilience coping [9], and students transitioning to college during the pandemic had flatter CARs compared to those who did so pre-pandemic [10]. Although cortisol reactivity is a healthy response to stress, repeated exposure to heightened levels of cortisol can lead to wear and tear on the body [11].

To our knowledge, the current body of research has not examined the effect of the pandemic -related restrictions on cortisol reactivity as a response to acute stressors. It has long been known that uncontrollability, novelty and social-evaluative threat (SET) can lead to an enhanced physiological response to stress [[12], [13], [14]]: Although it is unlikely to include elements of SET, re-emerging from lockdowns into an environment where things are not quite as before and where one is expected to adhere to new social rules include both novelty and lack of control, and consequently may lead to stronger physiological responses to our environments. The current study allows us to test this hypothesis in a natural experiment.

The data were originally collected as part of an experimental study with a student sample in which we tested the effect of a mental arithmetic task with task-related payment variation on salivary cortisol. Approximately half of the data collected in the study were collected prior to the March 2020 lockdown, and the remaining in November and December 2020 under restrictions. After the first lockdown, participants were typically returning to the lab and campus for the first time in more than six months, needing to negotiate novel lab restrictions and wider campus rules when attending the lab.

Although the original experiment was not designed to measure the impact of lockdown restrictions on anticipatory stress, given that those attending the experiment after November 2020 (post-lockdown) were more likely to experience social novelty and uncontrollability in navigating their way to the laboratory than those attending in March 2020 (pre-lockdown), the data was used as a natural experiment to provide timely evidence about the impact of the pandemic. Notably, these differences in novelty and uncontrollability occurred before the experiment, as participants arrived at the lab, rather than during the experiment itself which remained identical before and after lockdown. Consequently, this paper will focus primarily on differences in the initial, baseline sample. We hypothesised that cortisol levels would be higher on arrival at the lab in November 2020 than they were in March 2020.

## 2 Method

### 2.1 Participants

One-hundred and sixty-one university students took part in the study. Seventy-five of the participants took part in the study in March 2020 before the first lockdown and 86 participants took part in November and December 2020 when the lab was able to re-open (under restrictions). As suggested by previous research [15], six participants were excluded due to having raw cortisol values > 4 SDs from the mean, and three participants were excluded for not completing a demographic questionnaire in the main study, leaving a total of 152 participants. Sensitivity analyses are described below to check the robustness of findings with and without outliers. All conditions and data exclusions are reported. As the data from the experimental manipulation is reported elsewhere, a number of measures were recorded but will not be reported here.^1^ Ethical approval was granted by the University of Aberdeen School of Medicine, Medical Sciences and Nutrition (CERB/2019/12/1831).

### 2.2 Materials and procedure

All participants were taking part in an experiment to examine the cortisol response to a computer-based mathematical task (results to be published elsewhere). The experiment took place on-campus at the University of Aberdeen, in a single computer lab with 20 computers, within the Economics Department. In line with all in-person experimental stress paradigms, adaptations were required during the pandemic [16]. Although the experiment protocol remained the same across both groups, the participants in the post-lockdown group had to navigate a number of additional health- and safety restrictions before and during the experiment. This included changes to the standardised invitations (see Supplemental Material), staggered arrival to the lab, a skin temperature reading, completing a health questionnaire prior to entering and wearing a face covering. In addition to this, social distancing, reduced building capacity and a one-way system was enforced throughout campus and the lab. For example, the lab had capacity for 20 participants but after the initial lockdown this was reduced to 8 participants to always allow for 2-m social distancing. Finally, both experimenters were required to wear full personal protective equipment (PPE), including a lab coat, gloves, face covering and apron, in the post-lockdown sessions.

Although the focus here is on the novel restrictions in place prior to the experiment starting, cortisol samples were collected at four points during the experiment (see Fig. S1 in Supplemental Material).

The first part of the experiment consisted of a 10-min relaxation phase during which participants were offered colouring pens and colouring sheets to relax. Directly after this phase, participants provided a baseline saliva sample using Cortisol Salivettes® (Sarstedt, Nümbrecht, Germany), which is the main sample focused on in this analysis. Thereafter, participants completed a 10-min experimental task (incentivised maths calculations), followed by two further 10-min relaxation phases. Each phase was followed by provision of another saliva sample, resulting in one pre-task and three post-task samples. All experiments took place at 2pm. Participants were asked to avoid eating, drinking alcohol, smoking, sleeping, brushing their teeth or performing vigorous exercise in the 2 h prior to the experiment. During the experiment but after the first (baseline) sample, participants were randomly allocated to one of three payment conditions in the work task; a fixed payment of £5 for solving a minimum of 10 questions and otherwise no payment, £0.20 per correct answer or £0.60 per correct answer. There was a maximum of 50 questions that could be solved and all participants were paid a show-up fee of £7.50. After the initial 10-min relaxation phase, participants completed the General Health Questionnaire (GHQ-12; [17]. After the work task, participants completed a short demographic questionnaire, and a self-report checklist to indicate any activities or medications, including hormonal contraceptives, that may affect HPA axis activity.

### 2.3 Analysis

Saliva samples were stored in a freezer at −20C until all were ready for analysis. Samples were analysed for cortisol content using the DELFIA assay (a time-resolved fluorescent immunoassay; Biochemisches Laboratory, University of Trier). The intra-assay was 5.40%. Raw cortisol samples were transformed using a natural logarithmic transformation due to the skewed distribution of the samples to avoid violating any statistical assumptions [15] and then we carried out two-tailed t-tests comparing the pre-task cortisol sample between the pre- and post-lockdown groups. In addition to this, cortisol output (AUCg) and cortisol response (AUCi) were calculated using the standard formulae [18] with all four cortisol samples and compared with t-tests. The primary outcome for the present study was the first cortisol sample taken, which was collected after arrival at the lab but prior to the experimental condition stress task, meaning it was not confounded by the stress task itself. As such, we conceptualise this first sample as an indicator of the end-point of the physiological stress experienced while navigating novel lockdown restrictions in the process of getting to the lab. For completeness, all post-task cortisol levels are reported.

The GHQ-12 was computed using its Likert scoring method (from 0 to 3). All analyses were carried out in R using the base package. For all tests, alpha was set at 0.05, and effect sizes were examined with 95% confidence intervals. In sensitivity analyses we also tested for group differences in cortisol markers after controlling for potential sample differences between pre-lockdown and post-lockdown using multiple linear regressions. As the lockdown group comparison was not planned prior to data collection, a priori sample size calculations were not carried out. However, using the critical t-value provided by a power analysis in G*Power [19], the minimal statistically detectable effect using the current sample was d=0.32.

## 3 Results

Of the 152 participants included in the analysis, the majority were female (*n* = 85, 55.92%), between 18 and 20 years old and doing an undergraduate degree (see Table 1).

**Table 1:** Study characteristics.

		Pre-lockdown	Post-lockdown
		*n* = 72	*n* = 80
Self-reported distress	GHQ-12	1.06 (0.48)	1.09 (0.58)
Gender	Female	33 (45.83%)	52 (65%)
	Male	39 (54.17%)	28 (35%)
Age group	18–20	22 (30.56%)	53 (66.25%)
	21–23	23 (31.94%)	16 (20%)
	24–26	9 (12.50%)	6 (7.50%)
	27–29	9 (12.50%)	2 (2.50%)
	30+	9 (12.50%)	3 (3.75%)
Year of study	Undergrad	41 (56.94%%)	71 (88.75%)
	Postgrad	31 (43.06%)	9 (11.25%)
Area of study	Arts & Social Sciences	16 (22.22%)	25 (31.25%)
	Business School	26 (36.11%)	14 (17.50%)
	Life Sciences	8 (11.11%)	34 (42.50%)
	Physical Sciences	22 (30.56%)	7 (8.75%)
Cortisol (nmol/L)	Sample 1	3.76 (1.95)	5.42 (3.24)
	Sample 2	3.62 (1.84)	4.67 (2.53)
	Sample 3	3.45 (1.87)	4.15 (2.23)
	Sample 4	2.96 (1.48)	3.61 (1.79)
	AUCg	141.29 (68.26)	183.87 (98.15)
	AUCi	−9.30 (36.15)	−33.12 (45.86)

*Note*: Frequency and means reported above. Proportion/standard deviation in brackets.

In a two-sided *t*-test examining our primary hypothesis, we found that Sample 1 cortisol was higher in the post-lockdown sample (*M* = 5.42 nmol/L, *SD* = 3.24) than the pre-lockdown sample (*M* = 3.76 nmol/L, *SD* = 1.95), transformed *t*(150) = 3.81, *p* < .001, *d* = 0.62, 95% CI: 0.29, 0.95). Sensitivity analyses that retained potential outliers (>4SD from mean) and participants with missing survey data showed no meaningful difference in results. There were some notable differences in study characteristics between the groups, such as the distribution of gender and age. To control for this and other characteristics which differed across the two groups, we carried out a multiple linear regression predicting the effect of lockdown group on cortisol controlling for gender, age, year, subject of study and self-reported use of medication or confounding activities which affect cortisol. The regression found that attending the lab post-lockdown remained a significant predictor of log-transformed Sample 1 cortisol (*β* = 0.22, *p* = .009, 95% CI: 0.06, 0.38). None of the control variables were significant predictors of cortisol. Including the aforementioned outliers did not impact on the significance of this result.

**Fig. 1:**
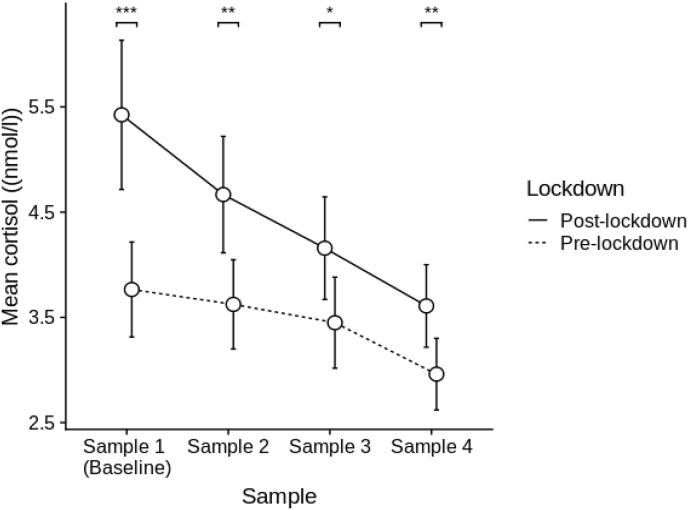
Raw cortisol with error bars showing 95% confidence intervals in the pre- and post-lockdown groups. ***p < .001, **p < .01, *p < .05

Additional analyses examining all samples found that the post-lockdown participants had significantly higher levels of cortisol across all samples taken (see Table 1 and Fig. 1): Sample 2, *d* = 0.47, 95% CI: 0.14, 0.80; Sample 3, *d* = 0.38, 95% CI: 0.05, 0.70; Sample 4, *d* = 0.43, 95% CI: 0.10, 0.75. Accordingly, AUCg was also higher in the post-lockdown sample (*M* = 183.87, *SD* = 98.15) compared to pre-lockdown sample (*M* = 141.29, *SD* = 68.26), *t*(150) = 3.07, *p* = .003, *d* = 0.50, 95% CI: 0.17, 0.83. Both samples typically had downward trajectories of cortisol, suggesting that there was no cortisol response to the stress task,^2^ but AUCi was more negative in the post-lockdown group (*M* = −33.12, *SD* = 45.86) than pre-lockdown (*M* = −9.30, *SD* =36.15), *t*(150) = −3.53, *p* < .001, *d* = −0.57, 95% CI: −0.90, −0.25.

Multiple linear regressions found that the significant effects remained after controlling for sample characteristics and medications: AUCg (*β* = 40.93, *p* = .016, 95% CI: 7.62, 74.24) and AUCi (*β* = −19.06, *p* = .021, 95% CI: −35.26, −2.86) (see Table S1 in Supplementary Materials).

## 4 Discussion

The present study employed a natural experiment to explore whether participants attending an experimental stress study post-lockdown arrived with elevated cortisol compared to those attending pre-lockdown. In summary, the results find that participants attending after lockdown had higher levels of cortisol on arrival, suggesting that the novel experience may have had an anticipatory effect on stress levels, eliciting a physiological response.^3^

Although the study did not include a self-report measure of acute anticipatory stress, there was no difference in “recent” psychological distress (GHQ-12; see Table 1) between the two groups. Furthermore, controlling for the GHQ-12 score in the regressions did not affect the relationship between lockdown group and any of the cortisol variables. Qualitative research has found that it can take many months for participants to return to “normality” after having to quarantine [20]. The current study adds to these findings, suggesting that the safety measures put in place when returning to normality may elicit additional stress to the extent that it affects the HPA axis.

Previous literature on cortisol reactivity have found heightened cortisol reactivity as a response to acute stress. For example, a recent study [10] found that minority students transitioning to college after the start of the COVID-19 pandemic exhibited stronger cortisol awakening response (CAR) than those who transitioned prior to the pandemic. In line with this, the current study found that the higher levels of cortisol in the post-lockdown group remained statistically significant throughout the experiment. However, although this could indicate heightened cortisol in general or heightened reactivity due to the stress of the pandemic, the size of the difference was much smaller than when comparing the two groups in the baseline sample. Indeed, AUCi demonstrated a steeper trajectory in the post-lockdown group and they did not find the task subjectively more stressful. This and the lack of difference in GHQ-12 score suggests that the difference seen between the groups in the latter samples is driven primarily by heightened stress upon arrival to the lab rather than generally higher levels of cortisol overall. However, as mentioned previously, the post-task samples should be interpreted with caution as there are multiple factors which may have affected them.

As the study described here was not originally designed to measure the effect of the COVID-19 restrictions, there are a number of potential limitations that need to be discussed. Firstly, pre-lockdown samples were stored at −20C for 14 months by the time of analysis, compared to the post-lockdown samples which were only frozen for 6 months. However, cortisol can be frozen at −20C for a year without detectable sample degradation [21] and it is likely that samples can be frozen for up to two years [15]. Secondly, the samples were collected at different points of the year. Although cortisol concentration has been shown to be statistically significantly different when comparing samples from February, March and April with samples taken in July and August [22], the current samples were taken in March and November, meaning that seasonal variation is unlikely to be the cause of the difference.

The study is a between-subjects design, rather than a within-subjects design. We also did not ask participants about their BMI or phase of menstrual cycle, both of which could affect stress reactivity [23]. However, if the increased anticipatory stress in the post-lockdown was due to higher BMI or the menstrual cycle phase in that sample, we would have expected to see heightened cortisol reactivity as a response to the stress task as well.

Another potential limitation is that the restrictions may have caused participation bias although we argue that it would disproportionately affect those who are the most stressed rather than the reverse, thereby underestimating the true effect. Finally, it would have been preferable to include survey measures of uncontrollability and novelty.

In conclusion, even if mental health is not generally poorer after periods of lockdown, there may still be acute stress associated with a “return to normal”, characterised by high levels of uncertainty. Although there has been a call for further stress research during COVID-19 [16], to our knowledge the impact of restrictions on physiological stress has not been considered. Future research should explore whether this is a short-term or long-term effect, as well as whether this is a unique feature of the COVID-19 lockdowns or if this is evident when navigating new procedures after more general major life events. Regardless, our findings suggest that lab-generated stress data provided in the period after the 2020 lockdown may be affected by acute stress. This should be taken into consideration when interpreting results.

## Conflict of interest statement

This manuscript has not been submitted to, nor is under review at, another journal or other publishing venue. The authors have no affiliation with any organization with a direct or indirect financial interest in the subject matter discussed in the manuscript.

## Footnotes

1 Post-task measures included self-reported stress, exhaustion, effort and strain during the stress task, all rated from 0 to 3 to maintain consistency with the GHQ-12 questionnaire in the pre-task questionnaire.

2 This is not unsurprising as the stressor task involved only a cognitive task rather than well-known physiological stressors, such as social-evaluative threat [12]. However, when responding to the item “After the task, how stressed do you feel?” participants rated themselves on average 1.30 (S.D = 0.88) on a scale from 0 to 3, indicating that there were some low levels of subjective stress, even if there was no physiological response.

3 As an additional analysis we compared the post-lockdown sample with a baseline sample from an earlier study (n = 137) carried out in autumn 2019 to see if there was a similar effect. Even though the earlier study had some differences in experimental protocol (including a repeated measures design and lower payment participation fees and incentives) which may have affected anticipation on arrival, a two-tailed *t*-test found that the effect of higher cortisol remained in the post-lockdown group (*M* = 5.42, *SD* = 3.24) than the earlier study baseline sample (*M* = 4.68, *SD* = 2.56) but that the difference was not statistically significant, *t*(215) = 1.68, *p* = .095, *d* = 0.24, 95% CI: −0.04, 0.52.
