# Supplementary material for: Salivary cortisol in university students after the COVID-19 pandemic
Source: Compr Psychoneuroendocrinol. 2022 Sep 20;12:100160. doi: 10.1016/j.cpnec.2022.100160 (PMC9487176; doi:10.1016/j.cpnec.2022.100160)
Supplement: Multimedia component 1 [file mmc1.docx]

**Supplementary materials**


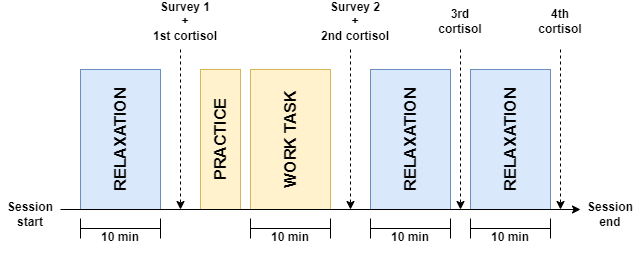


Figure S1. An overview of the experimental protocol

| Table S1. Multiple linear regressions predicting cortisol outcomes | | | | |
| --- | --- | --- | --- | --- |
|  |  | Baseline | AUCg | AUCi |
| (Intercept) |  | 1.59*** (0.11) | 156.79*** (23.15) | -7.18 (11.26) |
| Post-lockdown group | | 0.22** (0.08) | 40.93** (16.85) | -19.06* (8.19) |
| Male |  | 0.01 (0.08) | 11.17 (16.00) | 1.74 (7.78) |
| Age group | 21-23 years | -0.06 (0.09) | -5.22 (18.49) | 2.46 (8.99) |
|  | 24-26 years | -0.14 (0.16) | -24.84 (32.36) | 7.68 (15.74) |
|  | 27-29 years | -0.06 (0.18) | -5.07 (36.65) | 6.83 (17.83) |
|  | 30+ years | -0.22 (0.17) | -46.43 (33.51) | 7.24 (16.30) |
| Postgraduate |  | 0.08 (0.12) | 13.62 (25.16) | -0.69 (12.24) |
| Area of study | Business school | -0.08 (0.11) | -20.06 (22.03) | -3.57 (10.72) |
|  | Life sciences | 0.001 (0.10) | -18.23 (19.82) | -14.83 (9.64) |
|  | Physical sciences | -0.04 (0.12) | -13.60 (23.80) | -5.41 (11.57) |
| Self-reported variables that affect cortisol | Participated in activities | 0.04 (0.08) | 7.18 (16.19) | -9.26 (7.88) |
|  | Used medication | -0.16 (0.09) | -21.09 (18.98) | 4.83 (9.23) |
| *Note*: Reference categories are pre-lockdown group, female, age group 18-20 years, undergraduate, studying Arts & Social sciences, and have not participated in/used any confounding variables.  Baseline cortisol is log-transformed.  * p < .05, ** p < .01, *** p < .001 | | | | |

**Standardised invitation before March 2020**

Hello #fname# #lname#!

We wish to invite you to participate in an economics study in the Economics laboratory. PLEASE NOTE: we are looking ONLY for students who have not participated in the economics experiment “Work, Pay and Stress” before. If you attended one of these sessions last term, please do not register for this study.

To give you information about this study, you can access a participant information sheet by clicking the on the following link:

<https://www.abdn.ac.uk/business/documents/Study%202.1.pdf>

The list of available sessions is (please note that we use the US time format, i.e., month/day/year):

#sessionlist#

The experiment will last approximately 75 minutes. You will always be paid a participation fee of £7.50. At the experiment you will be randomly allocated to one of three groups in which you can earn additional fee: Up to £5, up to £10 and up to £30 depending on the group that you are allocated to.

If you would like to participate, you can sign up by clicking on the following link:

#link#

If there are any questions, please email us at the addresses below. Please do not reply directly to this email as it will go back to an unmonitored email account.

Best regards,

[REDACTED]

**Standardised invitation after November 2020 (changes in bold)**

Hello #fname# #lname#!

We wish to invite you to participate in an economics study in the Economics laboratory. PLEASE NOTE: we are looking ONLY for students who have not participated in the economics experiment “Work, Pay and Stress” before. If you attended one of these sessions last term, please do not register for this study.

To give you information about this study, you can access a participant information sheet by clicking the on the following link:

<https://www.abdn.ac.uk/business/documents/Study%202.1.pdf>

The list of available sessions is (please note that we use the US time format, i.e., month/day/year):

#sessionlist#

The experiment will last approximately 75 minutes. You will always be paid a participation fee of £7.50. At the experiment you will be randomly allocated to one of three groups in which you can earn additional fee: Up to £5, up to £10 and up to £30 depending on the group that you are allocated to.

If you would like to participate, you can sign up by clicking on the following link:

#link#

**Additional precautions have been taken due to the pandemic. The day before the experiment you will be contacted to confirm that you have not experienced any symptoms associated with COVID-19 (see bottom of this email). On the day of the experiment a researcher will meet you at the (INSERT ENTRANCE). It is important that you arrive at the agreed time and at the right entrance, otherwise you may not be able to take part. Prior to entering the lab we will need to take your temperature using an IR forehead thermometer. If you have any symptoms of COVID-19 you will be turned away.**

If there are any questions, please email us at the addresses below. Please do not reply directly to this email as it will go back to an unmonitored email account.

Best regards,

[REDACTED]

**Please do not take part in the study if you have experienced any of the following symptoms in the past 14 days:**

- **A high temperature/fever**
- **A new cough**
- **New loss of, or change to sense of smell or taste**

**And/or**

- **If you have received a letter advising you to Shield.**
